# Research on the performance of the active vibration cutting system for the rotary cylinder of a tunneling machine

**DOI:** 10.1038/s41598-023-44044-3

**Published:** 2023-10-05

**Authors:** Miao Xie, Yuqi Li, Rendong Nie, Zhixiang Liu, Jun Mao, Hongyu Zhang, Xinli Yu

**Affiliations:** 1 College of Mechanical Engineering, Liaoning Technical University, Fuxin, 123000 China; 2 College of Mines, Liaoning Technical University, Fuxin, 123000 China; 3 Xinjiang Key Laboratory of Intelligent Exploit and Control of Open-Pit Mine, Xinjiang, China

**Keywords:** Mechanical engineering, Coal

## Abstract

High-efficiency rock breaking technology is an important problem to be solved urgently in deep mining. The existing auxiliary rock breaking technology for coal mine excavation has problems such as polluting coal, high requirements for tool materials, and difficulty in subsequent washing. In this paper, an active excitation cutting system for rotary cylinder of cantilever roadheader based on alternating valve is proposed. According to the classical calculation formula of cutting load of roadheader, considering the stiffness-damping characteristics of cutting part and cutting cylinder, the simulation algorithm of cutting load is compiled based on MATLAB simulation analysis software. The excitation cutting experiments of different cutting depths are carried out on the cutting test bench, and compared with the simulation algorithm of cutting load. Taking the cutting load as the discriminant index, the influence of oil supply pressure and excitation frequency of rotary cylinder on the cutting load is analyzed based on the simulation algorithm of cutting load. The results show that the error between the simulation results and the experimental results of the active excitation state is less than 12%, and the two are in good agreement. Under the condition of 30 HZ, when the excitation amplitude is 8 Mpa, 10 Mpa, 12 Mpa, 14 Mpa, when the rotary cylinder excitation amplitude is 10 Mpa, the minimum is 131.42 KN. Compared with the rotary cylinder excitation amplitude under the condition of 10 Mpa, when the excitation frequency is 30 HZ, 35 HZ, 40 HZ, 45 HZ, when the excitation frequency is 40 HZ, the minimum is 83.08 KN, indicating that changing the excitation amplitude or oil supply pressure of the rotary cylinder is helpful to adjust the cutting performance.

## Introduction

Every year, over 12,000 km of new tunnels are excavated in Chinese coal mines, of which 30% are hard rock excavation tunnels^1^. Due to factors such as mining environment, underground working space, and operation technology, the excavation speed of rock tunnels is less than 100 m/month^2^. In addition, the commonly used cutting teeth of traditional coal mine tunnel boring machines are difficult to break hard rock and have a large load, and the cutting teeth and tooth seats are prone to damage and fall off, requiring frequent maintenance and replacement. Underground applications have proven that the cutting and rock breaking method of cutting teeth is difficult to achieve efficient excavation of hard rock tunnels^3^.

Since the nineteenth century, scholars around the world have developed many new rock breaking methods, such as disc cutter oscillation cutting (ODC), high-pressure water jet rock breaking, liquid nitrogen assisted rock breaking, microwave rock breaking, chemical expansion agent rock breaking, laser rock breaking, and other methods. Kovalyshen^4^ and Dehkhoda^5^ established a mechanical model for rock breaking through disc cutter vibration cutting, and pointed out that ODC technology can reduce the cutting load of the disc cutter, but its power required for rock breaking will not decrease. Karekal et al.^6^ studied the influence of parameters such as disc cutter oscillation frequency and rock strength on cutting force, and compared various traditional disc cutting methods to demonstrate the superiority of this technology in hard rock excavation operations. Liu Fanyong et al. proposed a water jet pre, mid, and post assisted rock breaking method ^7^, and with the help of a rock cutting test bench and a high-pressure water jet rock breaking system, studied the characteristics of the impact of pulse jet pre made cracks on the cutting load and tool temperature of the cutting teeth under different nozzle structures and working environment conditions^8^. Oh et al.^9^ conducted a study on the rock cutting depth model based on the kinetic energy of abrasive jet, and found that there is a power function relationship between the cutting depth and the maximum kinetic energy. Dehkhoda et al.^10,11^ conducted a study on rock damage under the action of pulse frequency and length, and found that the generation of cracks is mainly caused by pulse frequency, and its propagation is influenced by the size of pulse length. The cavitation bubbles formed on the impacted surface are also caused by pulse frequency. Dai et al.^12^ found that liquid nitrogen jet has a good effect on improving rock cutting efficiency, and the combined effect of thermal shock and jet impact is beneficial for crack propagation and rock cutting efficiency. Hassani and Nekovaght^13^ designed a microwave assisted rock breaking equipment and tested the actual effect of microwave assisted rock breaking, exploring the crushing characteristics of different rocks under different microwave powers. Boev et al.^14^, Maker et al.^15^, Cho et al.^16,17^ studied the possible electrical breakdown process in rocks. The results indicate that the impact stress wave does indeed act on the surrounding rock, leading to “internal damage” to the rock. When the impact of shock stress waves on the rock exceeds the strength of the rock itself, the rock will be destroyed. The high-pressure gas fracturing rock breaking technology uses corresponding devices to achieve a supercritical state of liquid carbon dioxide. When the pressure inside the pipe reaches the rated pressure, the supercritical carbon dioxide instantly changes phase, releasing a large amount of high-pressure gas at the injection end, and fracturing rock^18^ is carried out through high-pressure gas. Laser rock breaking is the process of rapidly heating the surface of a rock using a high-energy laser beam, causing an instantaneous increase in local rock temperature and generating local thermal stress. Due to the different thermal expansion coefficients and melting points between mineral particles, intergranular and intragranular fractures occur within the rock, and may even induce mineral particles to transition from solid to molten and gaseous phases, forming high-temperature plasma, and then using auxiliary airflow or other methods to break the rock, It is a non-contact physical rock breaking method^19^. Reed et al.^20^ proposed a systematic drilling plan by conducting laser drilling experiments on different rocks. Bjorndalen et al.^21^ studied technical parameters such as the divergence angle, main lobe radius, and spot power density of laser beams, as well as basic issues such as the heat transfer characteristics of rock surfaces after laser irradiation, mathematical description of temperature fields, and their distribution patterns. The results indicate that the penetration rate of laser through rocks is mainly related to the average power density of the laser beam and the physical performance parameters of the rock material. The cutting methods mentioned above have greatly improved the mining efficiency compared to traditional mechanical cutting, but these methods are not applicable to each working condition^22,23^. As for laser cutting, its irradiation energy range is difficult to control, which can easily lead to physical damage to coal blocks. For example, water jet in jet mode may exacerbate the viscosity of viscous coal rock, and abrasive jet may cause abrasive particles to be doped into coal blocks, which increases the difficulty of subsequent coal washing and preparation.

The key purpose of the above methods is to reduce the difficulty of mining hard coal and rock. Therefore, the author proposes a rotary oil cylinder active vibration cutting system in this direction. By changing the hydraulic system principle of the excavation equipment, the cutting teeth can undergo reciprocating impact transportation driven by the oil cylinder. The impact effect of the cutting teeth is used to improve the coal crushing efficiency, reduce the cutting impedance of hard coal and rock, and thus improve the efficiency of hard coal mining. By using the flexibility coefficient method to establish the three-dimensional vibration equation of the yaw cutting condition, a cutting load simulation algorithm program is developed, and the effectiveness of the algorithm calculation results is verified using experimental results. Furthermore, the changes in cutting load under different fuel supply pressures and excitation frequencies are analyzed.

### Mathematical model of cutting system

#### Composition and working principle of the active vibration cutting system of the oil cylinder

**Figure 1:**
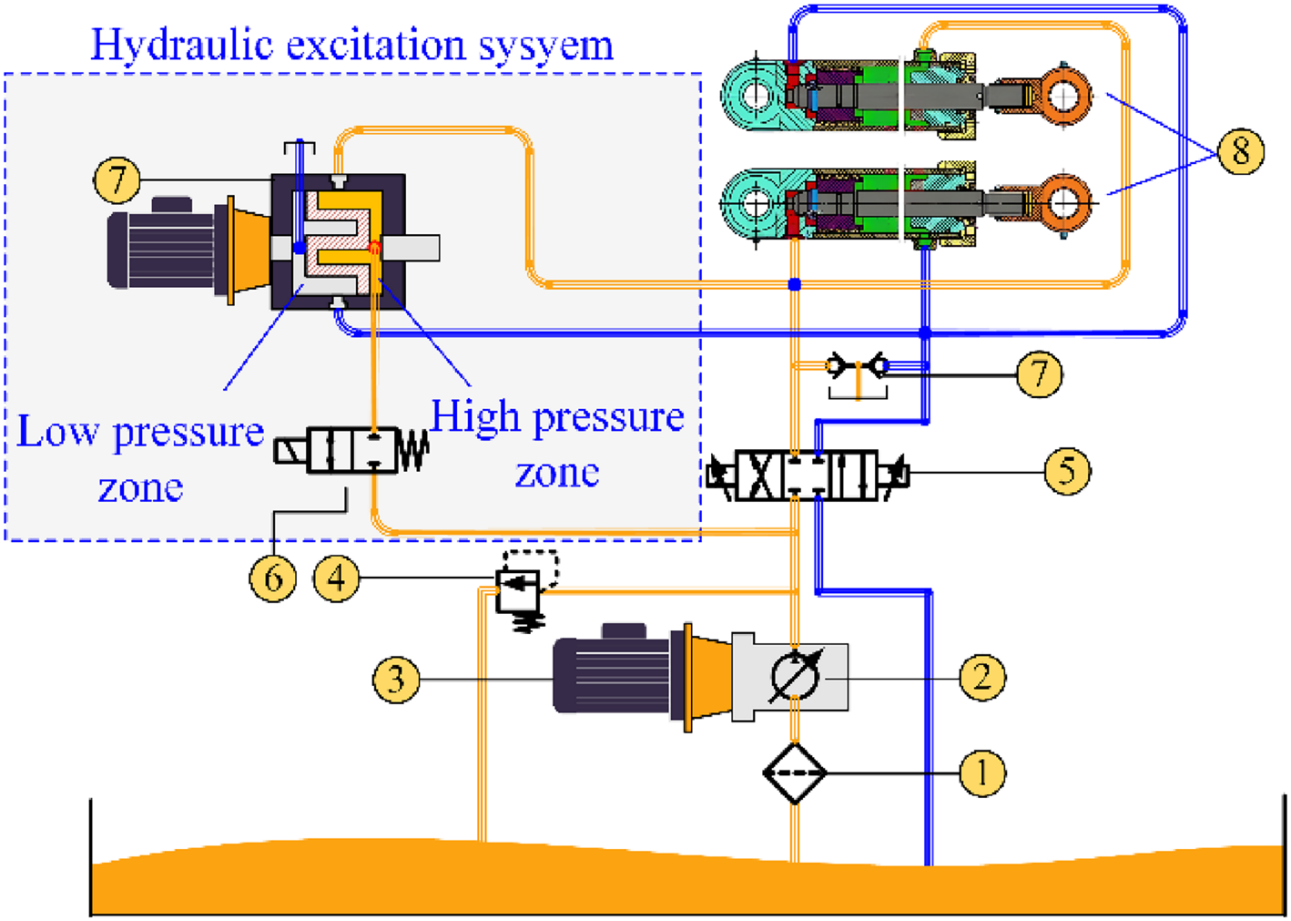
Principle diagram of active excitation cutting system.

The active vibration cutting system of the rotary oil cylinder is shown in Fig. 1, consisting of 1-filter, 2-variable pump, 3-pump station motor, 4-overflow valve, 5-three-position four-way electromagnetic directional valve, 6-two position two way electromagnetic directional valve, 7-alternating flow valve, 8-one-way valve, 9-rotary hydraulic cylinder, etc.

**Figure 2:**
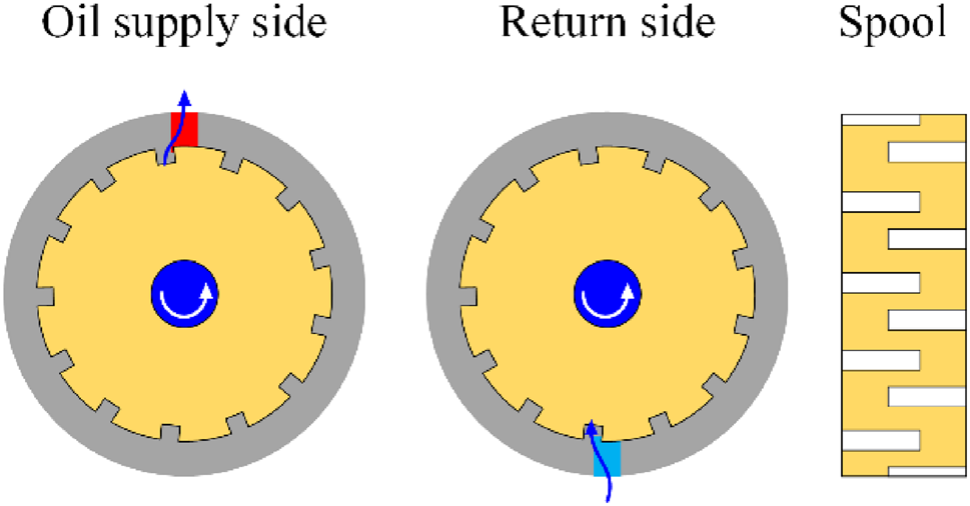
Schematic diagram of alternating flow distribution valve flow.

When the two two-way electromagnetic directional valve is opened, the excitation system takes effect. Its working principle is that the excitation source of the system is the alternating distribution valve, as shown in Fig. 2. Multiple oil grooves are staggered and evenly distributed around the valve core of the alternating distribution valve. The valve core achieves the excitation process through the alternating flow of the oil groove and the valve body. The valve can complete the oil supply and return^24^ every time the valve rotates one oil groove angle. As shown in Fig. 2, the characteristics of alternating supply and return sides of the alternating flow valve are utilized. The oil groove on one side of the shoulder of the valve core of the alternating flow valve inputs high-pressure oil from the pump station through a pipeline into the rodless chamber of the hydraulic cylinder. At the same time, the oil from the rodless chamber of the hydraulic cylinder returns to the oil tank through the other side of the alternating flow valve, which is the stroke of the cutting oil cylinder; When the alternating flow valve rotates to a certain angle, the high-pressure oil input from the pump station enters the rod cavity side of the cutting cylinder. At the same time, the oil without the rod cavity of the cutting cylinder returns to the oil tank through the alternating flow valve. This is the return stroke of the cutting cylinder. After repeated changes in stroke and return, the cutting cylinder will generate periodic vibration, achieving the excitation effect on the cutting head.

### Vibration model

**Figure 3:**
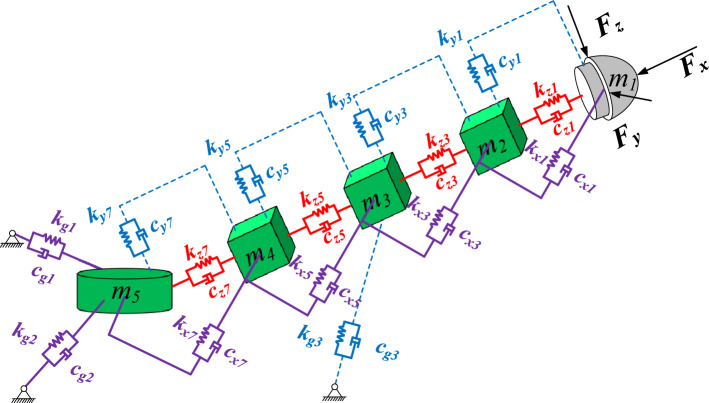
Three-dimensional vibration model of roadheader cutting unit.

Based on the structure and actual working state of the tunneling machine, the following assumptions are made: ① The load of the cutting head is the main excitation source, and the machine body does not move during the cutting process. ② The turntable is a rigid body. The three-dimensional vibration model of the cutting part of the tunneling machine is shown in Fig. 3, where:${\varvec{m}}_{1} ,{\varvec{m}}_{2} ,{\varvec{m}}_{3} ,{\varvec{m}}_{4} ,{\varvec{m}}_{5}$—Quality of cutting head, front telescopic section, lifting cylinder link section, reducer (cutting motor) section, and rotary table; ${{\varvec{k}}}_{\mathbf{x}1}, {{\varvec{k}}}_{\mathbf{x}3}, {{\varvec{k}}}_{\mathbf{x}5}{, {\varvec{k}}}_{\mathbf{x}7}, {{\varvec{k}}}_{\mathbf{y}1}{, {\varvec{k}}}_{\mathbf{y}3}, {{\varvec{k}}}_{\mathbf{y}5}{, {\varvec{k}}}_{\mathbf{y}7}, {{\varvec{k}}}_{\mathbf{z}1}, {{\varvec{k}}}_{z3}, {{\varvec{k}}}_{z5}{, {\varvec{k}}}_{\mathbf{z}7}$—The stiffness coefficients in the X, Y, and Z directions between the cutting head and the front expansion section, the front expansion section and the lifting cylinder link section, the lifting cylinder link section and the reducer (cutting motor) section, and the reducer (cutting motor) section and the rotary table, ${{\varvec{k}}}_{\mathbf{g}1}, {{\varvec{k}}}_{\mathbf{g}2}$—The stiffness coefficient in the Y-direction between the connecting section of the rotary cylinder and the rotary table, ${{\varvec{k}}}_{\mathbf{g}3}$—The stiffness coefficient in the Y-direction between the lifting cylinder link section and the rotary table; ${c}_{\mathbf{x}1}, {{\varvec{c}}}_{\mathbf{x}3}, {c}_{\mathbf{x}5}{,\mathrm{ c}}_{\mathbf{x}7}, {c}_{\mathbf{y}1}, {{\varvec{c}}}_{\mathbf{y}5}{, {\varvec{c}}}_{\mathbf{y}7}, {{\varvec{c}}}_{\mathbf{z}1}, {c}_{z3}, {{\varvec{c}}}_{z5},{{\varvec{c}}}_{\mathbf{z}7}$—Damping coefficients in the X, Y, and Z directions between the cutting head and the front expansion section, the front expansion section and the lifting cylinder link section, the lifting cylinder link section and the reducer (cutting motor) section, and the reducer (cutting motor) section and the rotary table, ${{\varvec{c}}}_{\mathbf{g}1}, {{\varvec{c}}}_{\mathbf{g}2}$—Damping coefficient in the Y-direction between the connecting section of the rotary cylinder and the rotary table,${{\varvec{c}}}_{\mathbf{g}3}$—Damping coefficient in the Y-direction between the lifting cylinder link section and the rotary table.

### ${\varvec{X}}$ and ${\varvec{Z}}$ direction vibration model

According to Fig. 3, the vibration differential equations in the X and Z directions are:

1 $$ M\ddot{X} + C_{X} \dot{X} + K_{X} X = F_{X} $$

2 $$ M\ddot{Z} + C_{Z} \dot{Z} + K_{Z} Z = F_{Z} $$

where ${\varvec{M}} = diag\left[ {m_{1} ,m_{2} ,m_{3} ,m_{4} ,m_{5} } \right]$ is the mass matrix of the system; ${\varvec{F}}_{{\varvec{X}}} = \left[ {F_{x} ,0,0,0,0} \right]^{T}$ and ${\varvec{F}}_{{\varvec{Z}}} = \left[ {F_{z} ,0,0,0,0} \right]^{T}$ is external load excitation; ${\varvec{X}} = \left[ {x_{1} ,x_{3} ,x_{5} ,x_{7} ,x_{9} } \right]^{T}$ and ${\varvec{Z}} = \left[ {z_{1} ,z_{3} ,z_{5} ,z_{7} ,z_{9} } \right]^{T}$ is the displacement of the system; ${\varvec{K}}_{{\varvec{X}}} ,{\varvec{C}}_{{\varvec{X}}} ,{\varvec{K}}_{{\varvec{Z}}} ,{\varvec{C}}_{{\varvec{Z}}}$ represents the stiffness matrix and damping matrix of the system in both the direction and direction, respectively. The stiffness matrix is obtained by the flexibility coefficient method, and the damping matrix is given by the damping coefficient.

**Figure 4:**
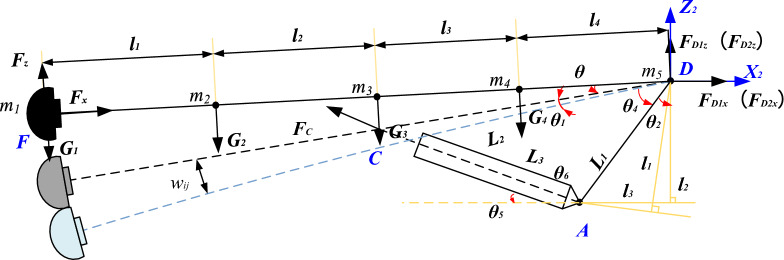
Deformation model diagrams in *X* and *Z* directions.

Deformation model diagrams in X and Z directions is shown in Fig. 4, where: ${\varvec{F}}_{{\varvec{C}}}$—Support force between the cutting lifting cylinder and the rotary table; ${\varvec{R}}$—The radius of the circle where the hinge point between the rotary cutting cylinder and the rotary table is located; ${\varvec{l}}_{1} ,{\varvec{l}}_{2} ,{\varvec{l}}_{3} ,{\varvec{l}}_{4}$—The distance between the cutting head and the front expansion section, the connection section between the front expansion section and the lifting oil cylinder, the connection section between the lifting oil cylinder and the reducer (cutting motor) section, and the distance between the reducer (cutting motor) section and the rotary table; ${\varvec{w}}_{{{\varvec{ij}}}}$—The deflection generated at point ${\varvec{m}}_{{\varvec{i}}}$ per unit force acting on point ${\varvec{m}}_{{\varvec{i}}}$.

To obtain the flexibility matrix of the system, it is necessary to divide the rotary table and cutting part into two parts. First, the force on the rotary table is solved, and then the stiffness of the cutting lifting cylinder is considered to determine the rotation angle of the cutting arm. Then, the deflection displacement at $m_{j}$ is calculated. According to Fig. 4, the force on the cutting lifting cylinder can be obtained as:

3 $$ F_{C} = \frac{{G_{1} L\cos \theta + G_{2} \sum\nolimits_{j = 2}^{4} {l_{j} \cos \theta + G_{3} \sum\nolimits_{j = 3}^{4} {l_{j} \cos \theta + G_{4} l_{4} - F_{z} L} } }}{{\sum\nolimits_{j = 3}^{4} {l_{j} \sin \theta_{5} \cos \theta } }} $$

Based on the previous assumption that the rotary table is a rigid body and the cantilever of the cutting part is an elastic body, the flexibility matrix of the system can be solved, and the vibration differential equation can be solved by combining the dynamic and static methods. If a unit force is applied at point j, the displacement caused at point $i$ can be expressed as:

4 $$ \delta_{ij} = \delta_{j} + w_{ij} $$

Among them, $\delta_{ij}$ is the deflection caused by the cantilever swing of the cutting part caused by the extension and contraction of the cutting rotary cylinder caused by applying a unit force at point j; $\delta_{j} = \mathop \sum \limits_{m = i}^{4} l_{m} sin\theta_{j}$, $i,j = 1,2,3,4,5$; $\theta_{j}$ can be calculated by Eq. (6).

5 $$ \theta_{j} = {\text{acos}}\left( {\frac{{\left( {l_{3} + l_{4} } \right)^{2} + l_{5}^{2} - H_{C0}^{2} }}{{2l_{5} \left( {l_{3} + l_{4} } \right)}}} \right) - {\text{acos}}\left( {\frac{{\left( {l_{3} + l_{4} } \right)^{2} + l_{5}^{2} - \left( {H_{C0} + \delta_{Cj} } \right)}}{{2l_{5} \left( {l_{3} + l_{4} } \right)}}} \right) $$

where $x$ direction $\delta_{j} = F_{{Cx{\text{j}}}} /k$, $z$ direction $\delta_{j} = F_{{Cz{\text{j}}}} \frac{\Delta V}{{\Delta P}}$,

$$F_{{Cx{\text{j}}}} = \left( {G_{1} Lcos\theta + G_{2} \mathop \sum \limits_{j = 2}^{4} l_{j} cos\theta + G_{3} \mathop \sum \limits_{j = 4}^{4} l_{j} cos\theta + G_{3} l_{4} } \right)/\mathop \sum \limits_{j = 3}^{4} l_{j} sin\theta_{5} cos\theta$$;

$$F_{{Cz{\text{j}}}} = \left( {G_{1} Lcos\theta + G_{2} \mathop \sum \limits_{j = 2}^{4} l_{j} cos\theta + G_{3} \mathop \sum \limits_{j = 4}^{4} l_{j} cos\theta + G_{3} l_{4} + \mathop \sum \limits_{m = j}^{4} l_{m} } \right)/\mathop \sum \limits_{j = 3}^{4} l_{j} sin\theta_{5} cos\theta$$。

Flexibility matrix $$\delta_{ij}$$:

6 $$ \delta_{ij} = \sum\limits_{m = i}^{4} {l_{m} } \sin \theta_{j} + w_{ij} $$

where:

$$w_{11} = \frac{{\left( {2G_{1} + 1} \right)\left( {l_{1} + l_{2} } \right)^{3} }}{6EI}\left( {l_{1} + l_{2} } \right) + \frac{{G_{2} l_{2}^{2} }}{6EI}(3l_{1} + 2l_{2} )$$, $$w_{12} = \frac{{G_{1} \left( {l_{1} + l_{2} } \right)^{3} }}{3EI} + \frac{{\left( {G_{2} + 1} \right)l_{2}^{2} }}{6EI}(3l_{1} + 2l_{2} )$$.

$$w_{21} = \frac{{\left( {G_{1} + 1} \right)l_{2}^{2} }}{6EI}(3l_{1} + 2l_{2} ) + \frac{{G_{2} l_{2}^{3} }}{3EI}$$, $$w_{22} = \frac{{G_{1} l_{2}^{2} }}{6EI}(3l_{1} + 2l_{2} ) + \frac{{\left( {G_{2} + 1} \right)l_{2}^{3} }}{3EI}$$, $$w_{44} = \frac{{\left( {G_{4} + 1} \right)l_{3}^{2} l_{4}^{2} }}{3EI}$$.

### ${\varvec{Y}}$—direction Vibration model

The vibration differential equation in the Y direction is:

7 $$ M\ddot{Y} + C_{Y} \dot{Y} + K_{Y} Y = F_{Y} $$

where: ${\varvec{F}}_{{\varvec{Y}}} = \left[ {F_{y} ,0,0,0,0} \right]^{T}$ is excited by external load; ${\varvec{Y}} = \left[ {y_{1} ,y_{3} ,y_{5} ,y_{7} ,y_{9} } \right]^{T}$ is the displacement of the system;

${\varvec{K}}_{{\varvec{Y}}}$ and ${\varvec{C}}_{{\varvec{Y}}}$ are the stiffness matrix and damping matrix of the system in the Y direction, respectively, and the stiffness matrix is obtained by the flexibility coefficient method, while the damping matrix is given by the damping coefficient.

Deformation model diagram in Y direction is shown in Fig. 3, where: ${\varvec{F}}_{{{\varvec{G}}1}} ,{\varvec{F}}_{{{\varvec{G}}2}}$—Support force between cutting rotary cylinder and rotary table; $\user2{ w}_{{{\varvec{yij}}}}$—The deflection generated at point ${\varvec{m}}_{{\varvec{i}}}$—per unit force acting on point ${\varvec{m}}_{{\varvec{i}}}$.

**Figure 5:**
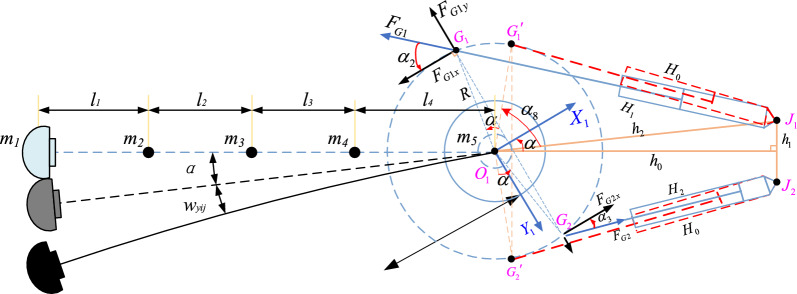
Deformation model diagram in Y direction.

To obtain the flexibility matrix of the system, it is necessary to divide the rotary table and cutting part into two parts. First, the force on the rotary table is solved, and then the stiffness of the rotary cylinder is considered to determine the rotation angle of the rotary table. Then, the deflection displacement at $m_{j}$ is obtained, According to Fig. 5, the force acting on the rotary cylinder can be determined as:

8 $$ F_{G1} = \frac{{F_{y} \left( {L - R\tan \alpha_{3} } \right)}}{{R\left( {\sin \alpha_{2} + \tan \alpha_{3} \sec \alpha_{3} \cos \alpha_{2} } \right)}} $$

9 $$ F_{G2} = \frac{{F_{y} \left( {L + R\tan \alpha_{2} } \right)}}{{R\left( {\sin \alpha_{3} + \tan \alpha_{2} \sec \alpha_{2} \cos \alpha_{3} } \right)}} $$

Based on the previous assumption that the rotary table is a rigid body and the cantilever of the cutting part is an elastic body, the flexibility matrix of the system can be solved, and the vibration differential equation can be solved by combining the dynamic and static methods. If a unit force is applied at point $j$, the displacement caused at point $i$ can be expressed as:

10 $$ \delta_{ij} = \delta_{yj} + w_{yij} $$

Among them, $\delta_{ij}$ is the deflection caused by the cantilever swing of the cutting part caused by the extension and contraction of the cutting rotary cylinder caused by applying a unit force at the $j$ point, $i,j = 1,2,3,4,5$.

11 $$ \delta_{yj} = \left( {R + \mathop \sum \limits_{m = i}^{4} l_{m} } \right)sin\alpha_{j} $$

12 $$ \alpha_{j} = {\text{acos}}\left( {\frac{{h_{2}^{2} - R^{2} - \left( {H_{1} + \delta_{Gj} } \right)^{2} }}{{2Rh_{2} }}} \right) - {\text{acos}}\left( {\frac{{h_{2}^{2} - R^{2} - H_{1}^{2} }}{{2Rh_{2} }}} \right) $$

where:$$\delta_{yj} = F_{{G1{\text{j}}}} \frac{\Delta V}{{\Delta P}}$$, and $$F_{{G1{\text{j}}}} = \left( {\mathop \sum \limits_{m = j}^{4} l_{m} - Rtan\alpha_{3} } \right)/R\left( {sin\alpha_{2} + tan\alpha_{3} sec\alpha_{3} cos\alpha_{2} } \right)$$。

Then the flexibility matrix $\delta_{ij}$ is:

13 $$ \begin{gathered} \delta_{ij} = \left( {R + \sum\limits_{m = i}^{4} {l_{m} } } \right)\sin \alpha_{j} + \frac{{\left( {\sum\limits_{m = i}^{4} {l_{m} } } \right)^{2} }}{6EI}\left( {3\sum\limits_{m = i}^{4} {l_{m} } - \sum\limits_{m = j}^{4} {l_{m} } } \right) \, \left( {i < j} \right) \hfill \\ \delta_{ij} = \left( {R + \sum\limits_{m = i}^{4} {l_{m} } } \right)\sin \alpha_{j} + \frac{{\left( {\sum\limits_{m = j}^{4} {l_{m} } } \right)^{2} }}{6EI}\left( {3\sum\limits_{m = j}^{4} {l_{m} } - \sum\limits_{m = i}^{4} {l_{m} } } \right) \, \left( {i \ge j} \right) \hfill \\ \end{gathered} $$

### Cutting load under lateral cutting condition of tunneling machine

#### Calculation of cutting load

**Figure 6:**
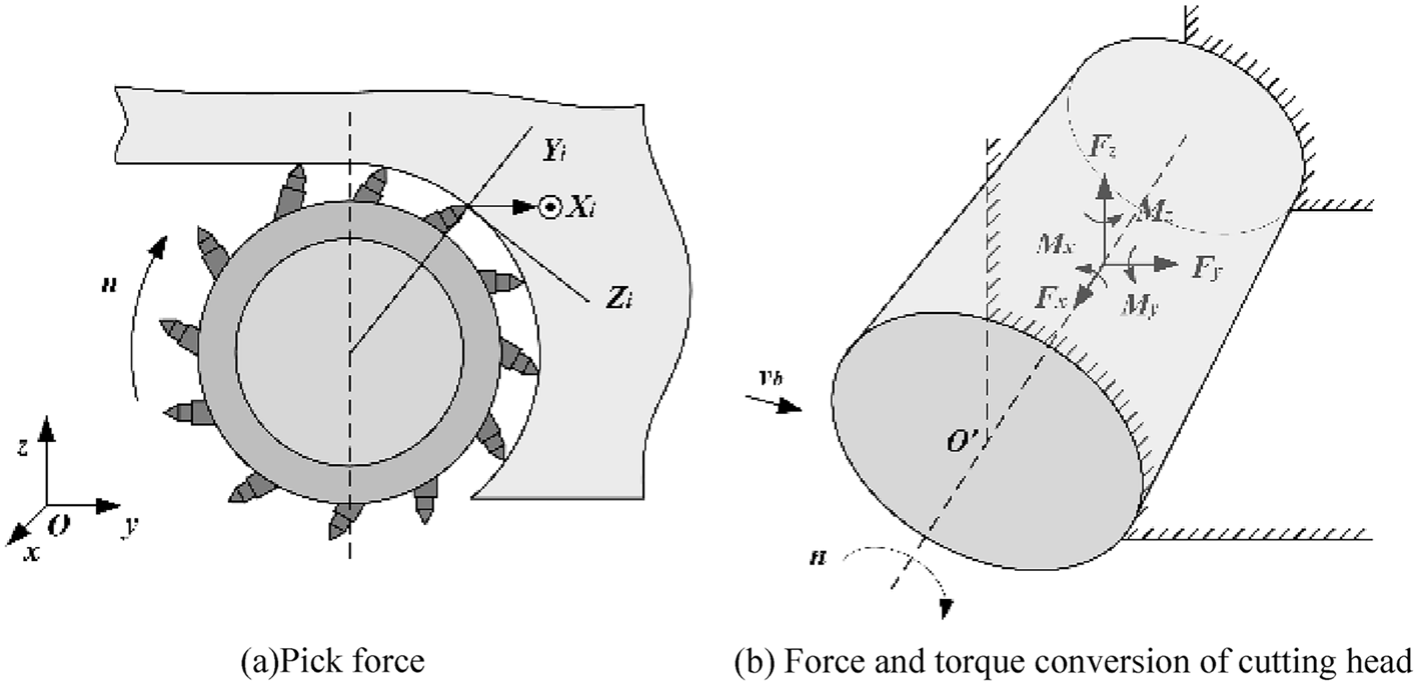
Roadheader cutting head force diagram under sweeping conditions.

According to the calculation method of instantaneous load on the cutting teeth^25^, calculate the cutting resistance $Z_{i}$, traction resistance $Y_{i}$, and lateral force $X_{i}$ of each cutting tooth (See Fig. 6a), and convert them to the coordinate transformation at the intersection point of the cutting plane where the corresponding cutting tooth is located and the cutting head axis (See Fig. 6b), to obtain the combined force of the cutting head along each coordinate axis, Among them,${v}_{b}$ is the lateral swing speed of the cutting part, and n is the cutting head speed, which is used to calculate the position and posture of the cutting teeth..

14 $$ Z_{i} = p_{k} \left[ {k_{t} k_{s} k_{y} \left( {0.25 + 0.018t_{d} h} \right) + 0.1S_{i} } \right] $$

15 $$ Y_{i} = Z_{i} \left( {0.15 + 0.00056p_{k} } \right)2.5/h^{0.4} $$

16 $$ X_{i} = Z_{i} \left( {\frac{{C_{1} }}{{C_{2} + h}} + C_{3} } \right)\frac{h}{{t_{d} }} $$

where: $p_{k}$ is the contact strength of rocks; $k_{t}$ is the type coefficient of the pick; $k_{s}$ is the comprehensive influence coefficient of the geometric shape of the pick; $k_{y}$ is the intercept influence coefficient; $t_{d}$ is the average intercept spacing; $h$ is the average cutting thickness; $S_{i}$ is the projected area of the blunt cutting edge in the traction direction; $C_{1} ,C_{2} ,C_{3}$ is the influence coefficient of the cutting diagram.

The combined forces of the cutting head along each coordinate axis under the transverse scanning condition are:

17 $$ F_{x} = \sum\limits_{i = 1}^{m} {X_{i} } = \sum\limits_{i = 1}^{m} {Y_{i} \sin \varepsilon_{i} } $$

18 $$ F_{y} = \sum\limits_{i = 1}^{m} {\left( {Z_{i} \cos \varphi_{i} + Y_{i} \sin \varphi_{i} \cos \varepsilon_{i} } \right)} $$

19 $$ F_{z} = \sum\limits_{i = 1}^{m} {\left( {Z_{i} \sin \varphi_{i} - Y_{i} \cos \varphi_{i} \cos \varepsilon_{i} } \right)} $$

where: $\varphi_{i}$ is the position angle of the $i$ pick at a certain moment;$r_{i}$ is the circumferential radius where the $i$ pick is located; $F_{x} ,F_{y} ,F_{z}$ is the cutting force exerted by the cutting head in the direction of the $X,Y,Z$ axis.

### Cutting load simulation algorithm

**Figure 7:**
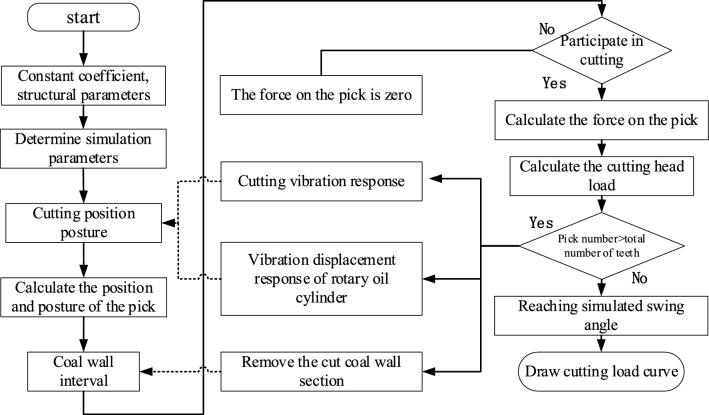
Cutting load simulation process.

Considering the impact of cutting vibration and rotary cylinder excitation on the variation of cutting load, based on the mathematical model and equation of the cutting system. Develop a MATLAB program to obtain a three-dimensional cutting load simulation program with or without active excitation under sweeping conditions. The simulation process is shown in Fig. 7.

*Step 1* Define and input the coefficients of the cutting load calculation equation, import the position and pose information of the cutting teeth, and determine the cutting head speed, cutting depth, drilling speed, pitch and swing speed, etc.

*Step 2* Determine the simulation duration and number of simulation steps, determine the starting and ending angles of rotation and pitch cutting, and determine the cross-sectional area of the roadway.

*Step 3* Calculate the total change in the swing angle of the cutting part and the total change in the position angle of the cutting head, determine the difference in the swing angle of the rotation based on the simulation steps, and determine the difference in the position angle of the cutting head based on the speed of the cutting head.

*Step 4* Determine the swing speed of the cutting part based on the structural parameters of the tunneling machine, simulation time, and swing angle variation.

*Step 5* Set the cyclic variables of cutting part swing angle and cutting head position angle, calculate the real-time cutting part swing angle and cutting head position angle.

*Step 6*: Determine the pose of each pick based on the pick pose equation, determine whether the pick participates in cutting, calculate the single pick load value for the picks participating in cutting, and summarize and import the result matrix.

*Step 7* Based on the vibration model in Sect. 1.2, use the cutting load to solve the change in the cutting part swing angle caused by three-dimensional vibration, and correct the real-time swing angle.

*Step 8* For the cutting teeth involved, use Boolean operation to remove the cut coal wall interval.

*Step 9* Repeat Steps 6 to 8 until the cutting swing angle reaches the set swing end angle.

When calculating the excitation state, it is necessary to calculate the excitation displacement response of the rotary cylinder in Step 7 and calculate the cutting position pose.

### Cutting experiment

#### Cutting experimental bench

**Figure 8:**
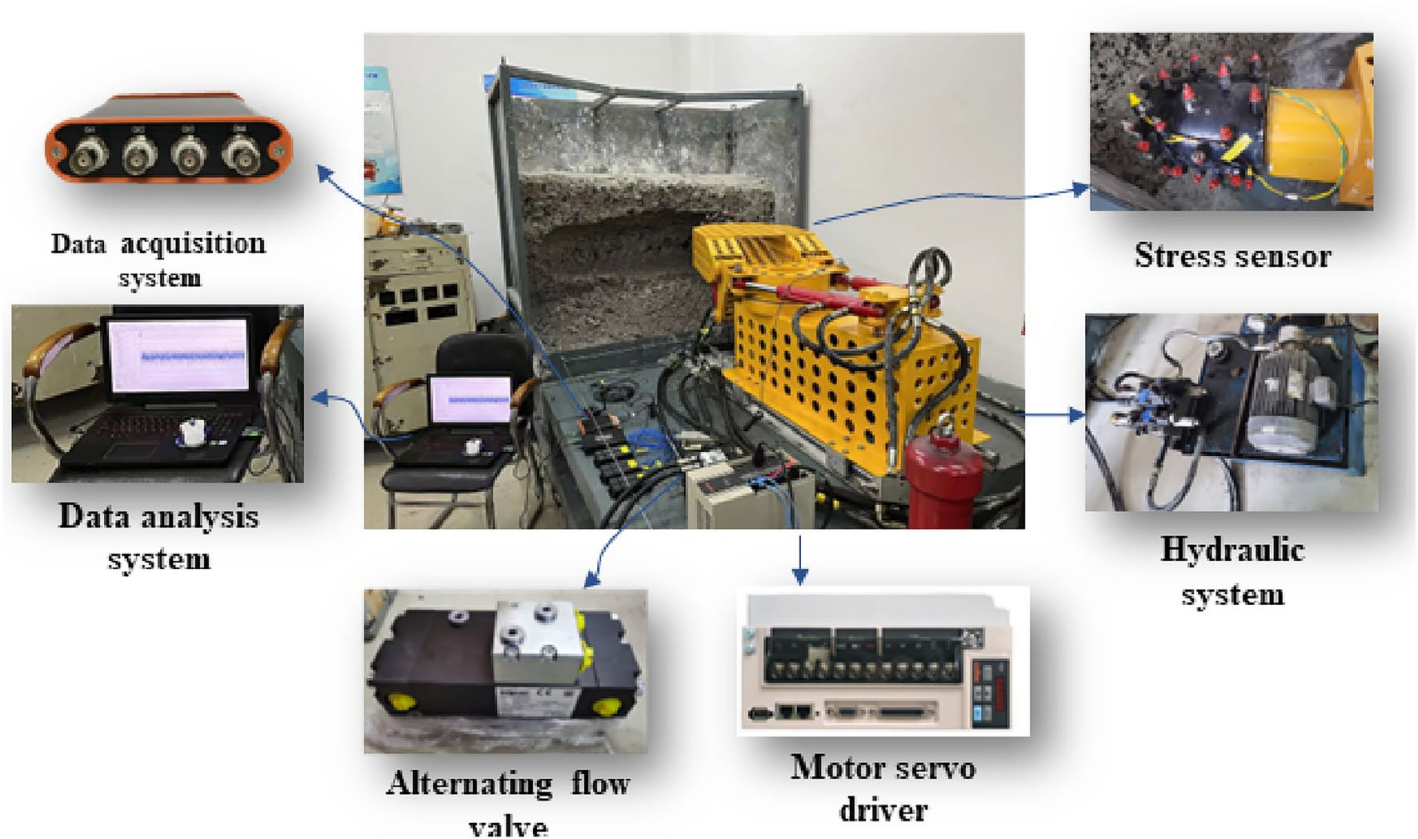
Simulated cutting experimental bench.

Based on previous experience in selecting similarity ratios for coal and rock cutting experiments and laboratory limitations, this article takes the EBZ230 tunneling machine as a prototype, with a similarity ratio of 1/3, and the experimental platform is shown in Fig. 8. The cutting experimental platform consists of a simulated coal wall, cutting section, cutting lifting cylinder, cutting rotary cylinder, main body, propulsion device, hydraulic power system, and alternating flow valve. The cutting power is 7.5 KW, the cutting head diameter is 350 mm, 41 cutting teeth are installed, and the cutting head body and tooth seat are made of 45 # steel. A micro force sensor is installed below the cutting teeth, with a maximum measurement accuracy of 3KN and a hydraulic system pressure of 10Mpa, The Dynaset HVB 350/9-40 model is selected for the alternating flow valve, and the main materials for simulating the coal wall include coal powder, cement, and water, with a ratio of 1.62:1:0.49.

The working principle of this experimental platform is as follows: after starting the hydraulic system, adjust the swing range of the cutting arm by adjusting the cutting rotary cylinder through the hydraulic directional valve, adjust the system pressure by rotating the relief valve pressure regulating screw, adjust the excitation frequency of the cutting system through the alternating flow valve, measure the cutting load value through the pin stress sensor installed inside the cutting teeth, and record the change process of the cutting load data through the data collection and analysis system.

This experiment takes the same cutting environment, cutting physical parameters, and motion parameters as the basic conditions, establishes two cutting methods without excitation and including excitation. The cutting rotary cylinder is used to make the cutting part rotate in the horizontal direction, and the alternating flow valve is used to control the lateral vibration of the cutting part. The influence of the active excitation cutting system of the rotary cylinder on the cutting contact force is compared and analyzed.

### Comparative analysis of simulation algorithms and experimental results

**Figure 9:**
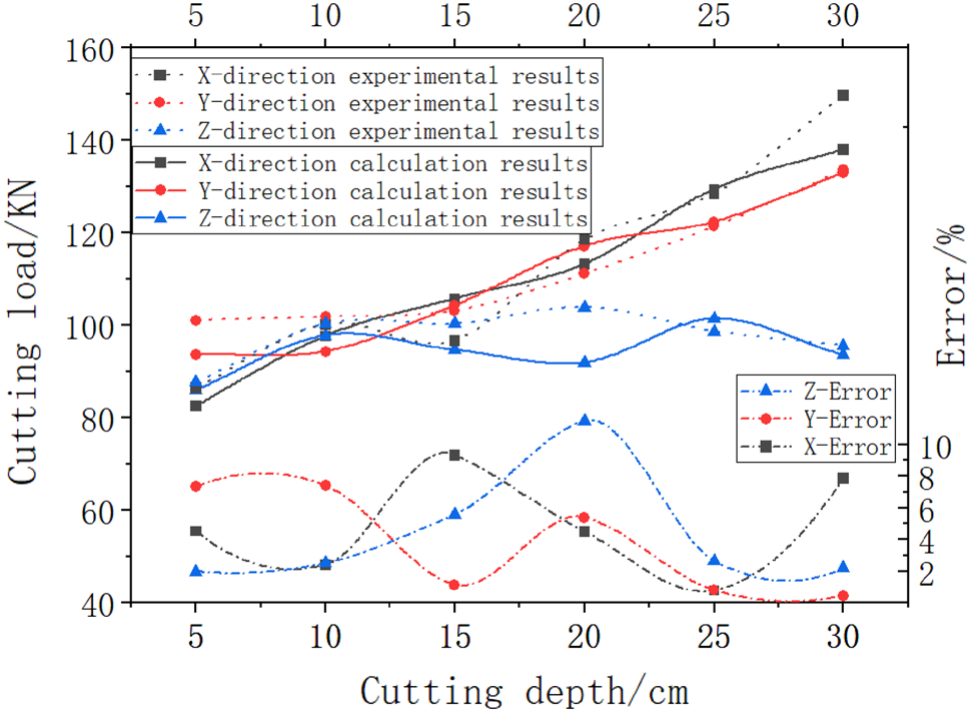
Comparison and analysis of simulation algorithms and experimental results.

To verify the effectiveness of the cutting load simulation algorithm, this article uses a cutting experimental platform to complete cutting experiments under six working conditions of cutting depth 5cm, 10cm, 15cm, 20cm, 25cm, and 30cm. The simulation cutting algorithm is used to calculate the cutting load under the same working condition, and the data from the cutting experiment and simulation cutting calculation method are compared and analyzed. Considering that there are significant differences in the number of unloaded cutting teeth under different working conditions, in order to eliminate the influence of random factors, This article only counts the cutting load data in the top 5% peak range, and the comparative analysis results are shown in Fig. 9.The other parameters are as follows: excitation frequency 30 HZ, oil supply pressure 10Mpa, rock contact strength $p_{k} = 485$ MPa, pickaxe tooth $k_{t} = 1.50$, comprehensive influence coefficient $k_{s} = 1.98$ of the cutting tooth, influence coefficient $k_{y} = 0.63$ of the cutting angle, average intercept distance $t_{d} = 25\;{\text{mm}}$, projected area $S_{i} = 20\;{\text{mm}}^{2}$ of the blunt cutting edge in the traction direction, influence coefficient $C_{1} = 1.0$, $C_{2} = 0.1$, $C_{3} = 0.2$, of the cutting diagram, cutting head speed $n = 36r/min$, and yaw speed $v_{b} = 1$m/min.

Analysis shows that the cutting load of both the calculation and experimental results increases with the increase of cutting depth. The experimental results showed that the average peak values of the cutting load for the six cutting depths in the X direction were 86.56 KN, 100.17 KN, 96.73 KN, 118.63 KN, and 128.47 KN, respectively. The calculated peak values of the cutting load for the six cutting depths in the X direction were 82.63 KN, 97.75 KN, 105.74 KN, 113.29 KN, 129.49 KN, and 138.04 KN, respectively, with a maximum error of 9.31% under the 15cm cutting depth condition; The experimental results showed that the average peak values of the cutting load at six different depths in the Y direction were 101.07 KN, 101.85 KN, 103.18 KN, 111.15 KN, 121.37 KN, and 133.57 KN, respectively. The calculation results showed that the average peak values of the cutting load at six depths in the Y direction were 93.68 KN, 94.34 KN, 104.37 KN, 117.13 KN, 122.34 KN, and 132.98 KN, respectively, with a maximum error of 7.31% at a depth of 10cm; The experimental results showed that the average peak values of the cutting load for the six depths in the Z direction were 87.75 KN, 100.36 KN, 100.29 KN, 103.86 KN, 98.73 KN, and 95.66 KN, respectively. The calculation results showed that the average peak values of the cutting load for the six depths in the Z direction were 86.03 KN, 97.85 KN, 94.68 KN, 91.95 KN, 101.37 KN, and 93.57 KN, respectively, with a maximum error of 11.46% at a depth of 20 cm; Although there is a certain deviation between the experimental results and the calculated results, considering that the presence of pouring pores and uneven materials in the simulated coal wall of the experimental platform can lead to unstable experimental results, it can be proven that the cutting load simulation algorithm is effective.

## Discussion

The cutting performance evaluation indicators of cantilever tunneling machines generally include cutting load, load fluctuation, and cutting specific energy consumption^26^. The load fluctuation is usually represented by the load variation coefficient, which is the ratio of the standard deviation to the average value of the cut load data at different time points. The cutting specific energy consumption refers to the work done by the cutting head to cut a unit volume of coal and rock, and its magnitude can reflect the level of cutting efficiency. It is mainly affected by the cutting load, cutting distance, rock density, and rock debris quality^27^. Because the cutting distance, rock density, and rock debris quality simulated in this article are consistent, the cutting load and load variation coefficient are selected as evaluation indicators.

Considering the good consistency between the calculation results of the cutting load simulation algorithm and the experimental results, this article only analyzes the parameter analysis of the active vibration cutting system of the rotary cylinder in the X direction of the cutting load change.

**Figure 10:**
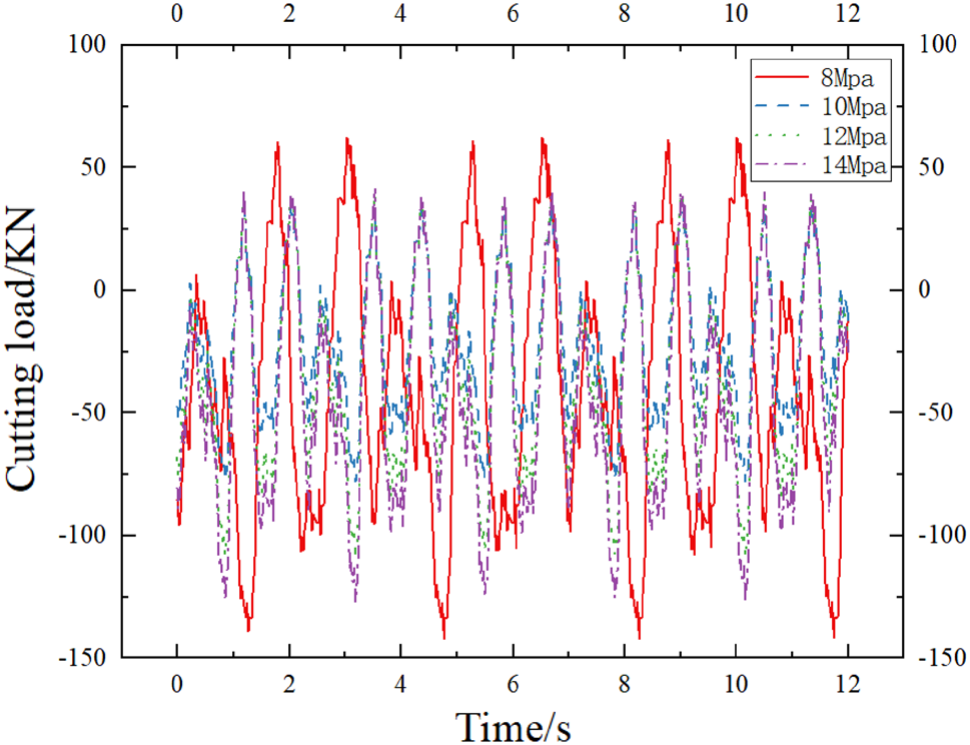
Cutting load variation curves under different excitation amplitudes.

The different changes in oil supply pressure of the active vibration cutting system under the excitation frequency of 30 HZ of the alternating flow valve are shown in Fig. 10. The horizontal axis represents the time of simulated cutting motion, and the vertical axis represents the change in cutting load in the X direction. From Fig. 10, it can be seen that the cutting loads at oil supply pressures of 8 Mpa, 10 Mpa, 12 Mpa, and 14 Mpa are 191.10 KN, 131.42 KN, 137.76 KN, and 156.01 KN, respectively, with load variation coefficients of 0.0615, 0.0908, 0.0515, and 0.0473. Compared with the cutting loads under different oil supply pressure conditions, the cutting load shows a trend of increasing from large to small with the increase of oil pressure. When the minimum cutting load is 10 Mpa, the maximum cutting load is 8 Mpa, When the fuel supply pressure is 10 Mpa, the cutting load is reduced by 31.23% compared to when the fuel supply pressure is 8 Mpa; The coefficient of load variation is maximum at 10 Mpa and minimum at 14 Mpa. The coefficient of load variation at 10 Mpa oil supply pressure is increased by 47.91% compared to that at 14 Mpa oil supply pressure. The reason for the analysis is that the cutting system is affected by changes in the hydraulic stiffness of the rotary cylinder, resulting in differences in the swing amplitude of the cutting arm. At 8 Mpa, ineffective cutting occurred, and at 10 Mpa, the cutting empty area increased, and the cutting load fluctuation increased. As the stiffness of the cutting rotary cylinder increased, the cutting disturbance decreased, and the load variation coefficient decreased.

**Figure 11:**
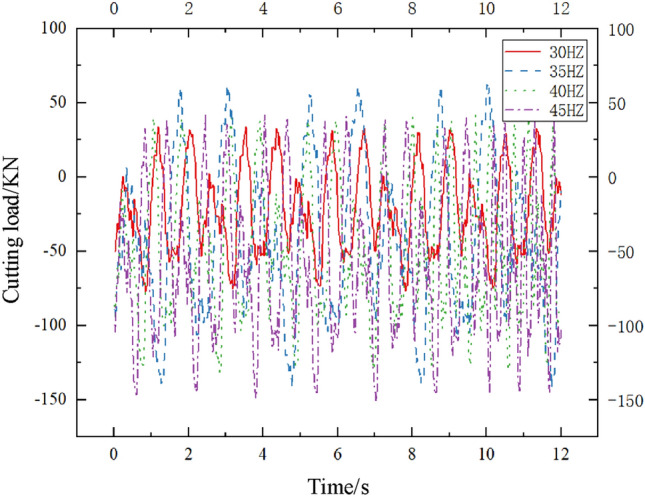
Cutting load variation curves under different excitation frequencies.

The variation of different excitation frequencies of the active vibration cutting system when the hydraulic system oil supply pressure is 10 MPa is shown in Fig. 11. The horizontal axis represents the time of simulated cutting motion, and the vertical axis represents the variation of cutting load in the X direction. When the excitation frequencies of the alternating flow valve are 30 HZ, 35 HZ, 40 HZ, and 45 HZ, the peak mean values of the cutting load are 131.42 KN, 96.91 KN, 83.08KN, and 92.12 KN, respectively. The coefficient of load variation is 0.0908, 0.01169, 0.0900, and 0.0840, respectively. Comparing the cutting load under different excitation frequency conditions, the cutting load shows a trend of changing from small to large to small as the excitation frequency increases. When the excitation frequency is 40 HZ, the cutting load is the smallest, When the excitation frequency is 30 HZ, it reaches its maximum, and the cutting load at 40 HZ is reduced by 36.83% compared to 30 HZ; The coefficient of load variation is maximum at an excitation frequency of 35 HZ and minimum at 45 HZ. The coefficient of load variation at an excitation frequency of 35 HZ is 39.17% higher than that at an excitation frequency of 45 HZ. The reason for the analysis is that as the excitation frequency increases, the vibration of the cutting teeth resonates with the coal and rock, leading to an increase in coal and rock cracks and a decrease in cutting load. At the same time, from the changes in cutting load and load variation coefficient, it can be seen that adjusting the excitation frequency can better improve cutting performance compared to adjusting the oil supply pressure.

## Conclusion

This article establishes a mathematical model for the active vibration cutting system of the rotary cylinder based on the proposed alternating flow valve. The cutting load simulation algorithm program is developed using MATLAB software, and the cutting load simulation algorithm is used to calculate the changes in cutting load under different oil supply pressure and vibration frequency conditions. The main conclusions are as follows:

1. A cutting load simulation algorithm was developed and compared with the simulation algorithm using a cutting experimental platform. It was found that the three-dimensional forces were 9.31%, 7.31%, and 11.46%, proving the effectiveness of the cutting load simulation algorithm;
2. By adjusting the cutting oil supply pressure, the cutting load can be effectively increased or decreased, showing a trend from large to small to large. When the oil supply pressure is 10Mpa, the cutting load is reduced by 31.23% compared to when the oil supply pressure is 8Mpa. At the same time, increasing the system oil supply pressure helps to reduce the fluctuation of cutting load;
3. By adjusting the cutting excitation frequency, the cutting load can be effectively increased or decreased, showing a trend from small to large to small. The coefficient of load variation at the excitation frequency of 35 HZ increases by 39.17% compared to the excitation frequency of 45 HZ. Adjusting the excitation frequency can better improve cutting performance compared to adjusting the fuel supply pressure.

## Data Availability

The dataset generated and analyzed during the current research period is not publicly available due to the unfinished project, but can be obtained from corresponding authors according to reasonable requirements.
